# Caffeine-augmented Sprint interval training outcomes in obese women: an examination by inter-individual analysis on physical and physiological adaptive responses

**DOI:** 10.3389/fnut.2025.1655449

**Published:** 2025-11-04

**Authors:** Xinying Zhao, Yang Liu

**Affiliations:** ^1^Institute of Physical Education, Xi’an Peihua University, Xi’an, Shaanxi, China; ^2^College of Sport and Art, Shenzhen Technology University, Shenzhen, Guangdong, China

**Keywords:** interval training, obesity, supplementation, strength, aerobic fitness

## Abstract

**Background:**

The objective of the present study was to elucidate the impact of a 12-week caffeine consumption before sprint interval training on the physical and physiological adaptations in obese women.

**Methods:**

Thirty overweight and obese women volunteered and were randomly divided into three groups: Caffeine (3 mg·kg^−1^ body mass, *n* = 10), Placebo (3 mg·kg^−1^ body mass in cellulose, *n* = 10), and Control (*n* = 10). Over 12 weeks, subjects in the Caffeine and Placebo groups consumed their supplements three times per week, ~60 min before each sprint interval training session. Fat mass, lower body strength, and cardiorespiratory fitness were evaluated at baseline and after the intervention. Fasting glucose, lipid profiles, and adipokines (leptin, adiponectin, and irisin) were measured 48 h before and after the intervention.

**Results:**

Both the Caffeine and Placebo groups exhibited significant improvements (*p* < 0.05) in these variables after 12 weeks of training. The Caffeine group showed significantly greater adaptive responses (*p* < 0.05) than the Placebo group in reducing fat mass (SMD = −0.27), enhancing strength (SMD = 0.30) and cardiorespiratory fitness (SMD = 0.43), and altering fasting glucose (SMD = −0.74), leptin (SMD = −0.23), adiponectin (SMD = 0.18), and irisin (SMD = 0.42) after the intervention.

**Conclusion:**

Supplementation of 3 mg·kg ^−1^ body mass caffeine before sprint interval training resulted in greater reductions in fat mass and improvements in strength and cardiorespiratory fitness, as well as more pronounced changes in fasting glucose and adipokines among overweight and obese women.

## 1 Introduction

The World Health Organization (WHO) has declared that obesity has nearly tripled since 1975 worldwide. Rates of overweight and obesity rates continue to rise, classifying obesity as a pandemic (1). While dietary intervention remains the most effective approach for weight loss (2), physical activity plays a key role in weight management and health improvement (3). It is recommended that individuals perform at least 150 min per week of physical activity to achieve clinically significant weight loss and improve overall health (3).

Various training methods improve performance, health, and reduce body fat (4–6). Among them, running-based high-intensity interval training (HIIT), including short sprints, has shown promising results (7). A common HIIT approach is sprint interval training (SIT), characterized by maximal-effort sprints interspersed with recovery (8, 9). While traditional SIT protocols often use 20–30 s sprints (e.g., running or cycling), emerging evidence indicates that short sprint bouts (<10 s) can produce greater training efficiency and meaningful adaptations (8, 9). Short SIT may be particularly suitable for overweight populations, as it requires less total training time, lowers perceived exertion, and increases enjoyment (8, 9) compared with longer interval formats. As a result, the implementation of short SIT protocol could be used as an effective approach to produce these benefits, minimize fatigue accumulation, and better accommodate participants to prolonged maximal efforts (8). Furthermore, this brief-duration of sprint intervention has been linked to improvements in VO_2max_ (9), reductions in inflammation (10), enhanced insulin sensitivity, and decreases in body adiposity (11).

Although exercise training is an effective strategy to stimulate post-exercise energy expenditure and promote fat loss and overall health (3), combining it with nutritional supplements that enhance post-exercise thermogenesis may enhance these benefits (12, 13). Caffeine, a widely consumed central nervous system stimulant (14), is known to enhance thermogenesis, fat oxidation (15), and weight management through increased secretion of noradrenaline and dopamine (16–18). Previous studies have shown that caffeine ingestion before exercise can elevate post-exercise energy expenditure by ~27% following 90 min of moderate-intensity exercise (17) and at intensities of 75% VO_2max_ (19). Short-term supplementation (1–8 weeks) may improve lipid profile, weight management, and performance by enhancing post-exercise energy expenditure and stimulating sympathetic nervous system activity and/or increasing the energetic cost of ventilation (13, 16, 18).

To optimize training adaptations in women, various doses of caffeine supplementation have been recommended to elicit adaptive responses that produce meaningful gains in physical performance and weight managements (12, 13, 17, 19). However, in female populations, the ingestion of approximately 3 mg·kg ^−1^ body mass appears optimal for improving performance (13, 16). Higher doses, such as 6 mg·kg ^−1^ body mass, have not demonstrated additional benefits and may be unnecessarily high for obese women (13, 17, 18). Therefore, selecting an appropriate dose is essential to enhance physical performance and health-related outcomes—including improvements in lipid profile and weight management—while minimizing side effects such as gastrointestinal discomfort (13). Consequently, integrating 3 mg·kg ^−1^ body mass caffeine intake with short SIT may be an effective strategy to enhance physical performance and weight managements. This approach could have significant practical implications for individuals seeking to accelerate fat reduction, improve physical performance, optimize lipid profiles, and modulate adipokines involved in blood glucose regulation and fat oxidation (18, 19). However, the specific effects of long-term caffeine supplementation combined with short SIT have not been investigated previously. Accordingly, the present study aimed to examine the effects of chronic caffeine ingestion, administered prior to short SIT sessions, on lipid profile, adipokine concentrations, performance adaptations, and fat loss in overweight and obese women.

## 2 Materials and methods

### 2.1 Ethics approval and study registration

The study involved healthy human participants and was approved by the Ethics Committees of the Xi’an Peihua University (EMRT, 2024.05.12/GT847) and was conducted in accordance with the most recent edition of the Declaration of Helsinki. All participants were given detailed information regarding the possible risks and discomforts associated with the study and were also required to sign informed consent agreements.

### 2.2 Sample size estimation and randomization

The sample size calculation was based on an effect size of 0.80 and a partial eta squared value of 0.21 from a previous study examining the effects of caffeine supplementation and HIIT on health-related factors in obese women (17). Using G*Power software (version 3.1.9, Universität Düsseldorf, Germany), we determined that a total of 30 participants was required to achieve 95% power at a significance level of 0.05 for F-tests, specifically mixed ANOVA (between-within factors). We allocated 10 women to each group. Randomization was performed using a computer-generated random number (simple randomization) in a 1:1:1 ratio to the Caffeine, Placebo, or Control groups. Group assignments were based entirely on chance and were unpredictable for both the authors and participants.

### 2.3 Participants

Participants were thirty overweight and obese women (BMI 25–40), sedentary (20), and low caffeine consumers (<50 mg/day) (21, 22) (Table 1). Before participating in the study, the subjects had to meet certain criteria. Firstly, they were required to have no previous injuries to their lower body. Additionally, their eligibility for training was determined by conducting screenings for any potential musculoskeletal, neurological, or orthopedic conditions that could have affected their ability to participate in the training. It was also important that none of the subjects used drugs or therapies for obesity, or had chronic diseases, endocrine disorders, or diabetes mellitus, as confirmed by an internal medicine specialist. Furthermore, the subjects had no history of physical activity prior to the start of the study and did not experience menstrual disorders such as dysmenorrhea, amenorrhea, or strong symptoms associated with premenstrual syndrome. To ensure compliance, a health-history questionnaire was administered during the participant recruitment process to screen for the use of nutritional, drug, and hormonal supplements. In addition, participants monitored their menstrual cycles and informed the investigator at the onset of menstruation. This information was used to schedule all baseline and experimental assessments during the late follicular phase, thereby controlling for potential hormonal and metabolic variations across the menstrual cycle. In accordance with the exclusion and inclusion criteria process, 22 participants were excluded from a total of 52, resulting in the inclusion of 30 participants in the study.

**Table 1:** Participant characteristics.

Characteristic	Caffeine (*n* = 10)	Placebo (*n* = 10)	Control (*n* = 10)
	Mean ± SD	Mean ± SD	Mean ± SD
Age (y)	22.1 ± 2.5	23.5 ± 2.8	22.8 ± 2.1
Height (cm)	165.6 ± 6.5	167.2 ± 4.7	164.6 ± 5.7
Weight (kg)	83.5 ± 5.6	85.8 ± 4.9	84.5 ± 4.4
BMI (kg.m^−2^)	30.7 ± 2.1	30.8 ± 2.2	31.2 ± 2.3

### 2.4 Experimental design

The current study is a longitudinal investigation with pre/post-test design with a duration of 15 weeks including: 1-week for familiarization with the training intervention and study aims, 1-week for pre-test, 12 weeks of training, and 1-week for the post-test. The participants underwent assessments of lower body muscular strength, cardiorespiratory fitness, and fat mass on two days [Day one (i.e., Monday); fat mass and strength, Day 2; (i.e., Wednesday) cardiorespiratory fitness] before and after the 12-week training period. Furthermore, blood samples were collected 48 h prior to and after the completion of the training period to evaluate resting adipokines (such as leptin, adiponectin and irisin) and lipid profiles (such as cholesterol, HDL, LDL, triglyceride), as well as fasting glucose levels in the morning. Both the Caffeine and Placebo groups participated in three non-consecutive days of training, specifically on Monday, Wednesday, and Friday in the afternoon at 4:00 p.m. while the Control group did not perform any physical activity programs and only continued their regular daily habits. Participants were instructed to adhere to their nutritional recommendations throughout the entire duration of the study.

### 2.5 Testing procedures

#### 2.5.1 Anthropometry

Body weight was measured to the nearest 0.1 kg using a digital scale (Tanita, Tokyo, Japan). Height was assessed to the nearest 0.5 cm with a wall-mounted stadiometer (Seca Model 214, Hamburg, Germany). Body fat mass (kg) was estimated using bioelectrical impedance analysis (Tanita Body Composition Analyzer, Tokyo, Japan). BMI was then calculated by dividing the participant’s mass in kg by the square of their height in meters.

#### 2.5.2 Lower body muscular strength

To evaluate lower body strength performance, the horizontal leg press exercise (Body-Solid, LVLP Leverage Horizontal Leg Press Machine) was employed. Lower body strength was estimated from submaximal lifts using the standard Kraemer and Fry procedure (22) and Brzycki equation (23). Prior to testing their maximum strength, participants engaged in a general warm-up including 10 min of light running and 10 min of stretching movements. During the strength test, the resistance was progressively increased in successive trials until the participants could no longer execute a proper lift with full range of motion and correct technique for five repetitions. Upon achieving a 5RM, the participants’ one-repetition maximum (1RM) was calculated using the following formula: 1RM = weight (kg)/1.0278 – (5RM × 0.0278) (23). This approach was selected because the 1RM test is highly strenuous which can be risky for individuals who are not trained in resistance training. During the testing procedure, spotters and researchers were present to offer assistance and ensure the safety of the participants.

#### 2.5.3 Cardiorespiratory fitness

After a warm-up of 10 min stretching and 5 min treadmill running (Technogym, Italy), participants performed an incremental treadmill test starting at 3 km·h−1, with speed increased by 2.5% every 2 min until volitional exhaustion. Test duration was used to estimate VO_2peak_ using the validated formula (24).


VO2peak(mL·kg1·min1)=1.444×Time(min)+14.99

#### 2.5.4 Blood sampling and analysis

Fasting blood samples (15 mL) were drawn from the antecubital vein into plain evacuated tubes to assess adipokines, lipid profiles, and fasting blood sugar. Samples clotted at room temperature for 25–30 min, were centrifuged at 1500 × g for 10 min, and serum aliquots were stored at −80 °C for later analysis. To control for circadian variation in hormones and biochemical markers, all blood draws were performed after a 10-h fast and 8 h of sleep, between 8:00 and 9:00 a.m. The photometric End Point method was employed to carry out the measurement of cholesterol, HDL, LDL, and triglyceride by available kits (Novus Biologicals, USA) using auto-analyser devices (Hitachi®, model 704, 902, Japan). For the measurement of glucose level, the ELISA kit (Eagle Biosciences, United States) was utilized. In addition, serum leptin and adiponectin (R&D Systems, Inc. Minneapolis, MN), as well as irisin (Phoenix Pharmaceuticals, Inc., Burlingame, CA) levels were evaluated by available ELISA kits in duplicate. The coefficient of variation (CV) was assessed to determine the intra-assay variance, which needed to be less than 5%.

#### 2.5.5 Control of diet and physical activity

Participants maintained usual diet and physical activity, documented 3-day food intake using COMP EAT 4.0 (~20% protein, 55% carbs, 25% fat), and abstained from alcohol, caffeine, and vigorous activity 24 h before testing. Throughout the 12-week intervention, adherence to baseline dietary habits was carefully maintained through scheduled personal interviews at follow-up visits and consistent daily reminders, supporting high compliance and limiting dietary variation that could influence study outcomes.

#### 2.5.6 Caffeine supplementation

Throughout the 12-week intervention period, participants in the Caffeine and Placebo groups received 3 mg.kg−1 caffeine (100% purity, Bulk Powders, United Kingdom) or placebo (cellulose), 1 h before training session, capsules identical in appearance (25). All participants were instructed to take the capsules with 100 cc of juice. It is important to highlight that the capsules were devoid of any labeling regarding their contents, ensuring that both the researchers and participants remained blind to the composition until the conclusion of the study. The placebo was designed to be visually and taste-wise indistinguishable from the active supplement and was provided in the same format. Participants were required to ingest their assigned supplements before each training session, with both the supplement and placebo distributed on a weekly basis. At the end of each week, participants were obligated to return all packets, regardless of whether they were used or not.

### 2.6 Training program

All participants maintained their regular physical activity routines. The Control group refrained from engaging in any structured physical activity programs and continued with their usual daily activities. In contrast, participants in the Caffeine and Placebo groups followed a training regimen specifically designed for obese women, consisting of 3x/week, 3 sets of 10 × 5-s sprints (i.e., all-out condition), 25-s recovery between sprints (i.e., self-paced running or walking), 3-min rest between sets (i.e., walking and stretching) for 12 weeks (8, 26). The choice of 5-s all-out sprints was informed by evidence that short-duration SIT protocols require less total training time, reduce perceived exertion, and increase exercise enjoyment compared with longer intervals (8, 9). Such attributes are particularly valuable in overweight and obese populations, where initial exercise tolerance, musculoskeletal load, and adherence may be limiting factors (19). Furthermore, brief supramaximal sprints help minimize excessive fatigue accumulation, allowing participants to deliver consistent maximal efforts and progressively adapt to high-intensity work without undue strain (8). Every training session began with a 15-min warm-up (including 5 min of running and 10 min of stretching and ballistic movements) and ended with a 10-min cool-down. All training took place on a wooden-court gym floor maintained at 27–28 °C and 45–55% humidity, under close supervision from a qualified strength and conditioning coach and a researcher to ensure adherence to the protocol.

### 2.7 Statistical analysis

Data are presented as mean ± SD. Normality was assessed using the Shapiro–Wilk test. A 3 (group) × 2 (time) ANOVA was used to detect significant differences. Significant differences were followed by Bonferroni post hoc tests while controlling for type I errors. Hedges’ g was used to calculate effect sizes (ES), which were categorized as trivial (< 0.20), small (0.20–0.60), moderate (0.60–1.20), large (1.20–2.00), or very large (> 2.00). The 95% confidence interval (CI) was reported as well (27). The significance level was set at 0.05. Individual percent changes (%Δ) were calculated as (post-pre)/pre × 100. CV (ratio of SD to the mean) and individual residuals (as the squared root of the squared difference between the individual and mean values) were used to evaluate inter-individual variability in responses to the intervention.

## 3 Results

Before the intervention, no significant differences were observed among groups (*p* > 0.05). All participants in the training groups attended all sessions and reported no injuries or caffeine-related side effects.

Following a 12-week intervention period, the Control group did not display any changes in the measured variables. In contrast, both the Caffeine and Placebo groups revealed significant differences when compared to the Control group across all variables (*p* = 0.001). Both the Caffeine and Placebo groups experienced significant changes (*p* = 0.001) in all variables post-intervention, with ESs ranging from trivial to large. Regarding the lipid profile, both the Caffeine and Placebo groups demonstrated similar changes after the training period, and no significant group by time interaction was observed between the training groups (*p* = 0.984–0.127; Table 2).

**Table 2:** Changes in lipid profile and fasting glucose from pre- to post-training for the groups (mean ± SD).

Variables	PRE	POST	Interaction effect	Hedge’s g (95% CI)	
HDL (mg/dL^−1^)					
Caffeine	46.7 ± 5.1	48.8 ± 5.4*		0.38 (−0.50 to 1.27)	Small
Placebo	47.3 ± 3.9	48.9 ± 4.5*	*p* = 0.001	0.36 (−0.52 to 1.25)	Small
Control	46.4 ± 5.4	46.5 ± 5.4		0.02 (−0.86 to 0.89)	Trivial
LDL (mg/dL^−1^)					
Caffeine	108.0 ± 8.5	102.3 ± 8.2*		−0.65 (−1.55 to 0.25)	Moderate
Placebo	105.6 ± 9.7	100.3 ± 8.0*	*p* = 0.001	−0.57 (−1.47 to 0.32)	Small
Control	104.9 ± 10.4	105.4 ± 10.2		0.05 (−0.83 to 0.92)	Trivial
Triglyceride (mg/dL^−1^)					
Caffeine	102.7 ± 7.6	97.3 ± 7.7*		−0.68 (−1.58 to 0.23)	Moderate
Placebo	101.3 ± 9.9	97.1 ± 9.6*	*p* = 0.001	−0.41 (−1.30 to 0.47)	Small
Control	100.5 ± 10.1	101.1 ± 9.8		0.06 (−0.82 to 0.93)	Trivial
Cholesterol (mg/dL^−1^)					
Caffeine	169.2 ± 11.3	158.4 ± 11.4*		−0.91 (−1.83 to 0.01)	Moderate
Placebo	170.7 ± 13.5	161.1 ± 12.6*	*p* = 0.001	−0.70 (−1.61 to 0.20)	Moderate
Control	170.9 ± 12.5	171.1 ± 12.0		0.02 (−0.86 to 0.89)	Trivial
Fasting glucose (mg/dL^−1^)					
Caffeine	102.6 ± 4.5	95.2 ± 3.0*†		−1.85 (−2.90 to 0.81)	Large
Placebo	101.5 ± 5.2	97.9 ± 3.9*	*p* = 0.001	−0.75 (−1.66 to 0.16)	Moderate
Control	100.4 ± 5.1	101.1 ± 5.5		0.13 (−0.75 to 1.00)	Trivial

*Denotes significant differences compared to PRE and Control. †denotes significant differences compared to Placebo.

**Figure 1:**
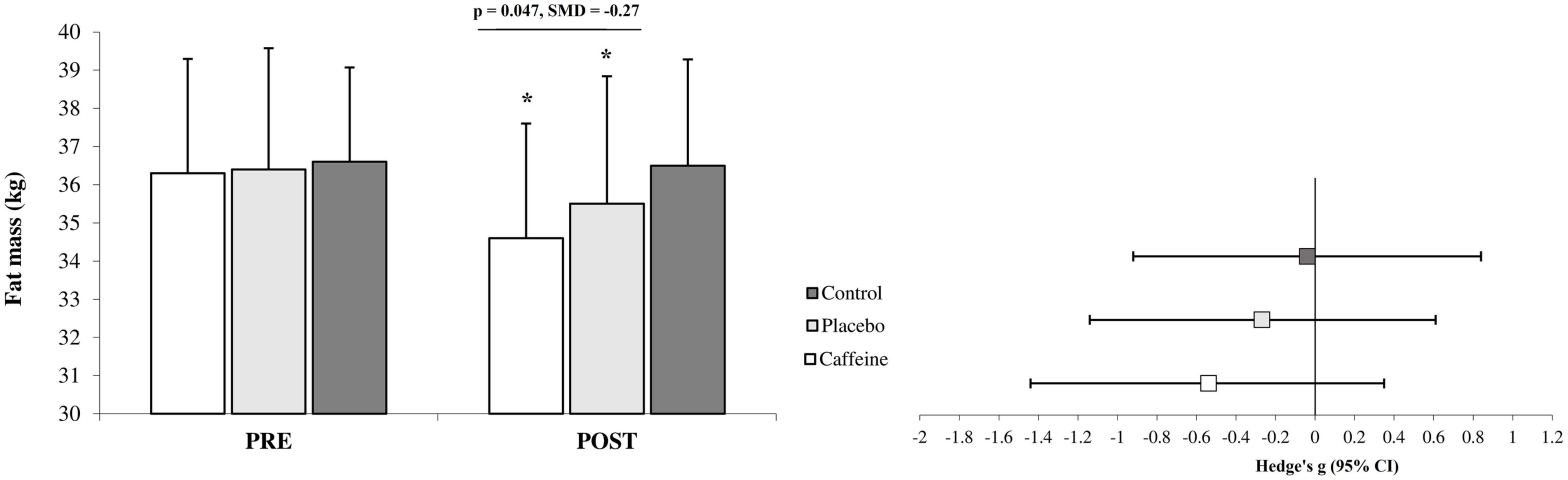
Changes in fat mass from pre to post-intervention in the groups (mean ± SD). *Significant differences vs. pre-intervention and Control (*p* = 0.001). Line indcates significant differences between Caffeine and Placebo group.

**Figure 2:**
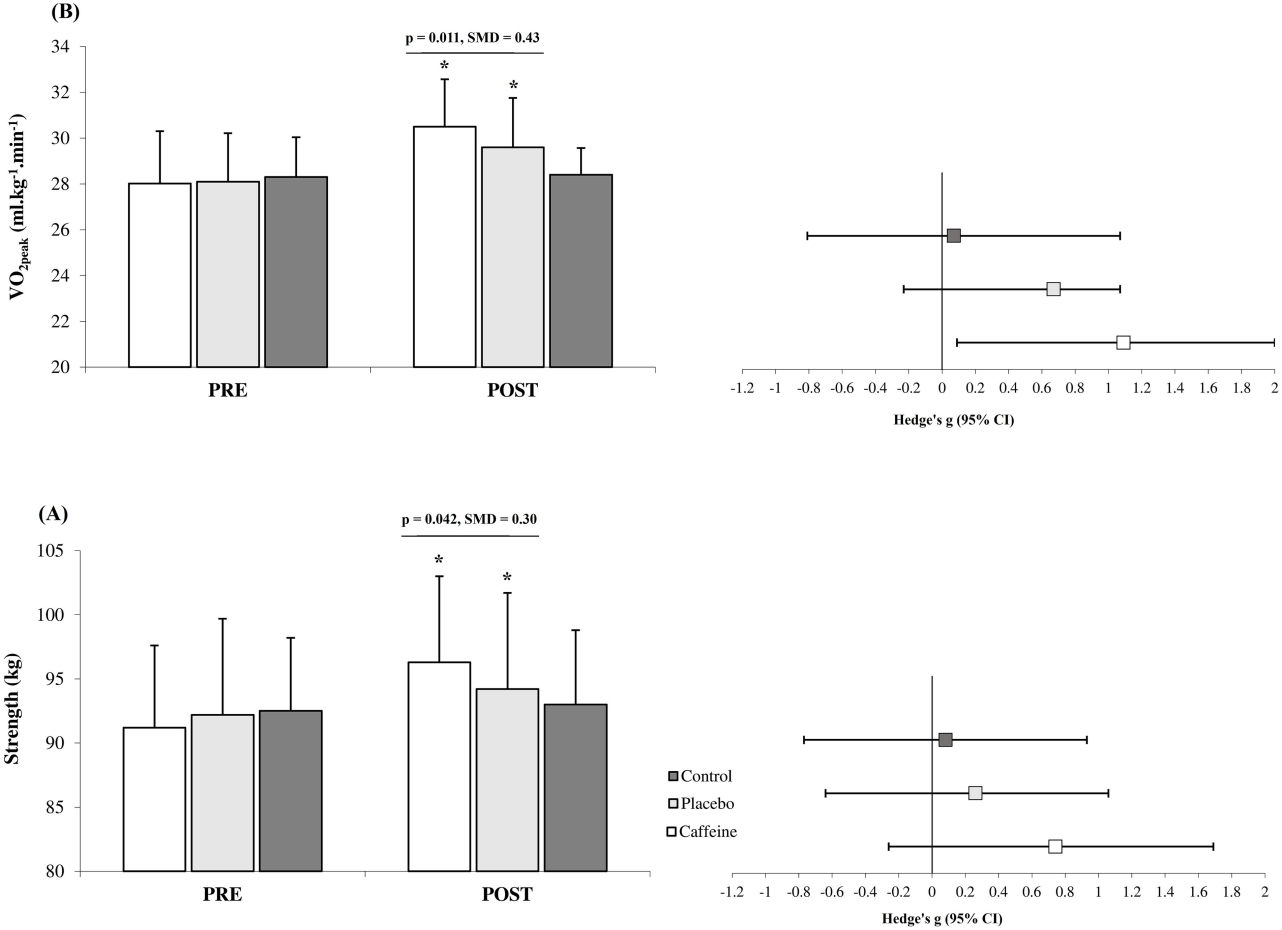
Changes in strength **(A)** and cardiorespiratory fitness **(B)** from pre to post-intervention in the groups (mean ± SD). *Significant differences vs. pre-intervention and Control (*p* = 0.001). Line indcates significant differences between Caffeine and Placebo group.

**Figure 3:**
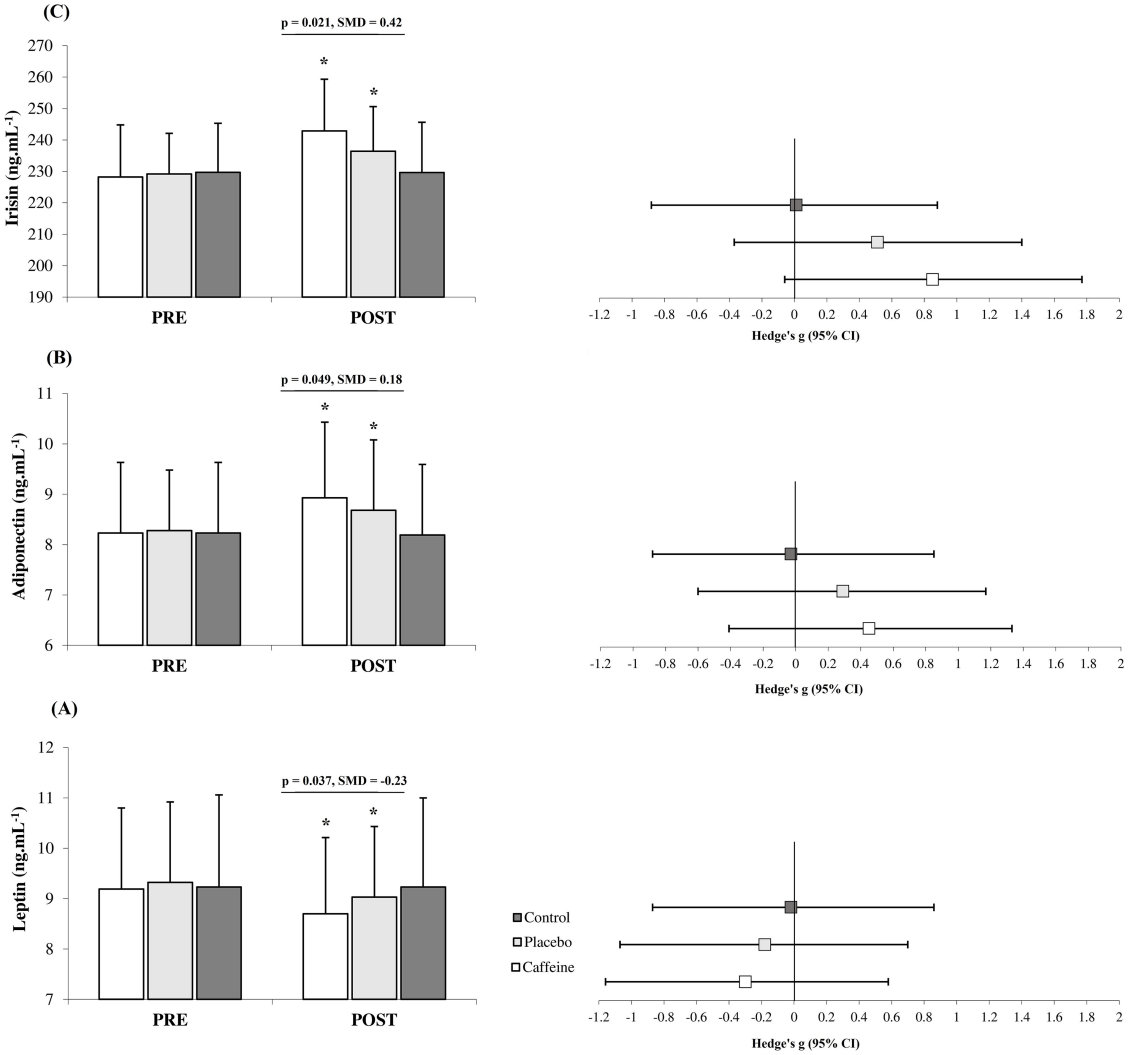
Changes in leptin **(A)**, adiponectin **(B)** and irisin **(C)** from pre to post-intervention in the groups (mean ± SD). *Significant differences vs. pre-intervention and Control (*p* = 0.001). Line indcates significant differences between Caffeine and Placebo group.

After 12 weeks, the Caffeine group showed more pronounced adaptations than the Placebo group across most measured variables including fat mass (standard mean difference (SMD) = −0.27, 95% CI = −1.15 to 0.61, Small difference, *p* = 0.047; Figure 1), strength (SMD = 0.30, 95% CI = −0.60 to 1.16, Small difference, *p* = 0.042; Figures 2A), VO_2peak_ (SMD = 0.43, 95% CI = −0.48 to 1.29, Small difference, *p* = 0.011; Figures 2B), fasting glucose (SMD = −0.74, 95% CI = −1.65 to 0.16, Moderate difference, *p* = 0.018; Table 1), leptin (SMD = −0.23, 95% CI = −1.10 to 0.66, Small difference, *p* = 0.037; Figures 3A), adiponectin (SMD = 0.18, 95% CI = −0.71 to 1.05, Trivial difference, *p* = 0.049; Figures 3B), and irisin (SMD = 0.42, 95% CI = −0.48 to 1.29, Small difference, *p* = 0.021; Figures 3C) after the 12-week intervention.

As shown in Table 3, the Caffeine group indicated more changes (i.e., ∆%) in fat mass, strength, VO2peak, fasting glucose, leptin, adiponectin, and irisin after the 12-week intervention and these changes were related to lower IRs in ∆% and CVs, which indicating more consistency in adaptive responses than the Placebo group.

**Table 3:** ∆% and inter-individual variability in the variable measured for the caffeine and placebo groups following the 12-week intervention.

Variables	∆%	IRs	CV (%)
Fat mass (kg)			
Caffeine	−4.7 ± 1.1*	0.96	−23.7
Placebo	−2.5 ± 1.4	1.11	−58.6
Strength (kg)			
Caffeine	5.5 ± 0.9*	0.77	16.6
Placebo	2.1 ± 1.4	1.15	66.2
VO_2peak_ (ml.kg^−1^.min^−1^)			
Caffeine	9.1 ± 2.9*	2.21	31.9
Placebo	5.3 ± 2.8	2.41	54.1
HDL (mg/dL^−1^)			
Caffeine	4.4 ± 1.7	1.45	38.2
Placebo	3.3 ± 2.3	1.70	69.6
LDL (mg/dL^−1^)			
Caffeine	−5.2 ± 1.5	1.15	−29.2
Placebo	−4.9 ± 2.5	1.85	−51.2
Triglyceride (mg/dL^−1^)			
Caffeine	−5.2 ± 1.3	1.05	−25.7
Placebo	−4.2 ± 1.5	1.14	−36.6
Cholesterol (mg/dL^−1^)			
Caffeine	−6.4 ± 1.5	1.25	−23.8
Placebo	−5.2 ± 2.1	1.35	−39.2
Fasting glucose (mg/dL^−1^)			
Caffeine	−7.1 ± 3.4*	1.82	−44.1
Placebo	−3.4 ± 3.6	2.06	−106.7
Irisin (ng/mL^−1^)			
Caffeine	6.4 ± 1.6*	1.37	25.8
Placebo	3.1 ± 1.8	1.41	59.4
Adiponectin (ng/mL^−1^)			
Caffeine	8.7 ± 1.7*	1.40	20.1
Placebo	4.6 ± 2.2	1.76	47.4
Leptin (ng/mL^−1^)			
Caffeine	−5.2 ± 2.3*	1.87	−44.1
Placebo	−2.8 ± 2.8	1.91	−100.1

*Significant differences compared with the placebo (*p* < 0.05).

## 4 Discussion

This study investigated whether short SIT combined with caffeine supplementation produces greater physiological adaptations than SIT with placebo in overweight and obese women. Over 12 weeks, both groups showed beneficial changes; however, consuming 3 mg·kg−1 caffeine one hour before each session led to greater fat mass reduction, strength and cardiorespiratory fitness gains, improved fasting glucose, and enhanced adipokine profiles. These findings suggest caffeine may potentiate SIT-induced adaptations, offering a practical, low-cost strategy to improve body composition and metabolic health in this population. Nonetheless, small effect sizes for some outcomes may limit their long-term clinical impact.

In line with previous studies, different forms of aerobic exercise—especially HIIT—are effective in reducing body fat among young women who are overweight or obese (11, 28). The reduction in fat mass after SIT may be due to elevated catecholamines and growth hormone, which trigger the breakdown of stored fat (lipolysis), along with increased mitochondrial biogenesis that enhances the body’s capacity to use fat for energy (29). In our study, caffeine intake before SIT yielded greater adaptations than placebo, likely due to its thermogenic effects (12, 17). Furthermore, caffeine may enhance fat loss by increasing energy expenditure, stimulating thermogenesis (15), and antagonizing adenosine receptors in muscle and the central nervous system, thereby elevating sympathetic activity (14, 30). As these pathways were not measured directly, they remain plausible mechanisms supported by prior evidence rather (11, 17, 28, 29) than confirmed causal factors.

Over 12 weeks, short SIT improved strength performance, though effect sizes were small, indicating modest benefits for enhancing strength gains. Similar benefits have been attributed to neuromuscular adaptations (31), while shorter interventions may be less effective; for example, Song and Deng (32) reported no strength gains after 6 weeks of SIT in basketball players, highlighting the roles of program duration and participant fitness level. SIT can enhance neuromuscular function through greater motor unit recruitment, improved intra- and inter-muscular coordination, and better storage and use of elastic potential energy (26). It may also induce muscular hypertrophy, contributing to strength gains (33). Moreover, the Caffeine group showed small ES but greater strength gains than the Placebo group, possibly due to enhanced muscle fiber activation and increased calcium release during exercise (13). It seems that caffeine consumption before training can influence both central and peripheral adenosine receptors, boosting central drive and reducing pain and fatigue sensations (14). It also increases serotonin release in the cerebral cortex, enhancing sympathetic activity while reducing inhibitory neural input (15, 17). These effects may heighten arousal during SIT, further stimulating muscle fiber recruitment and promoting greater strength adaptations (18).

**Figure 4:**
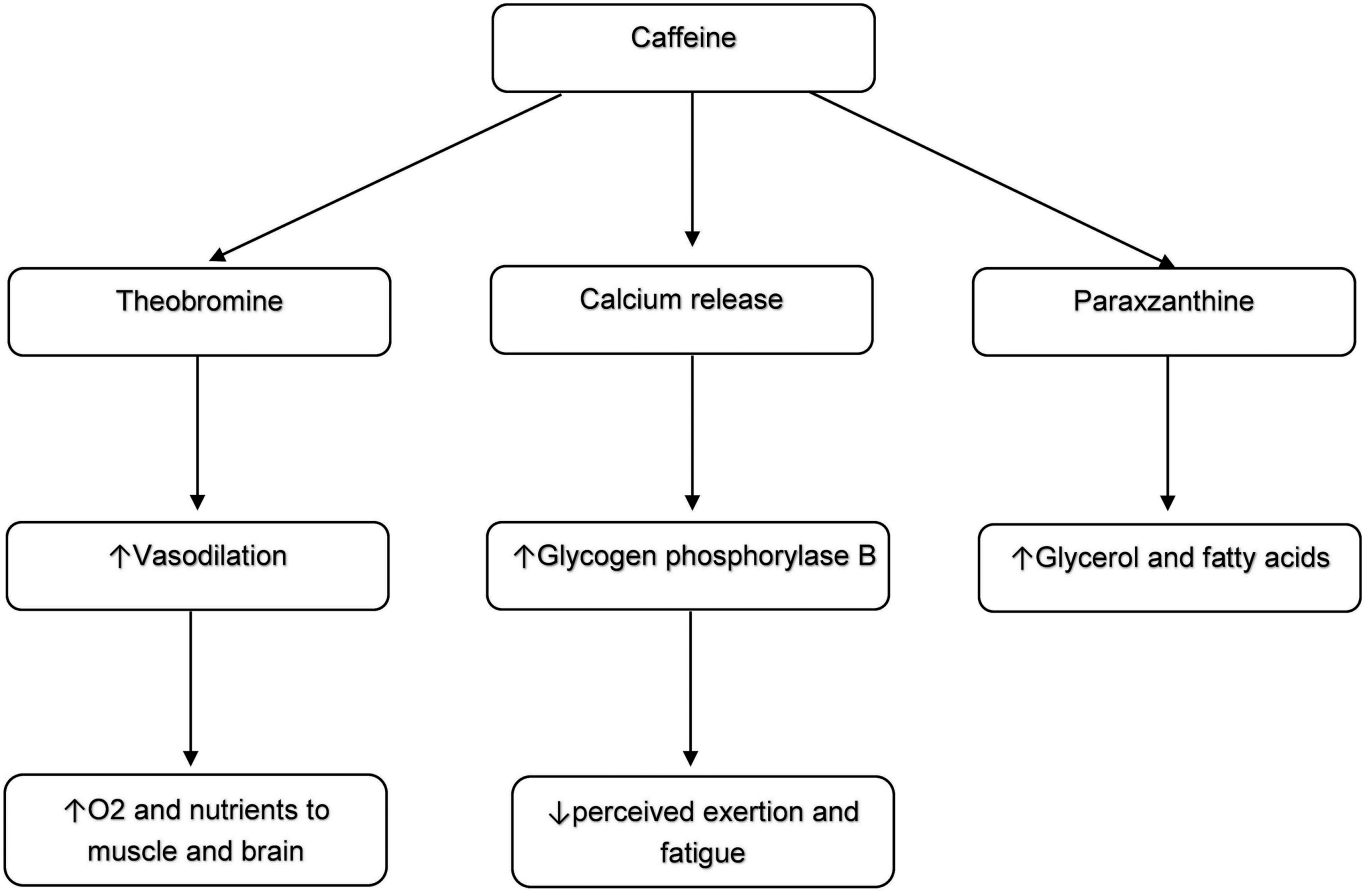
The mechanisms of caffeine in improving cardirespiratory fitness.

Both Caffeine and Placebo groups showed significant and moderate ESs in improving VO2peak, consistent with reports that SIT and short SIT improve aerobic fitness (5, 8, 9). In sedentary overweight and obese women, these gains likely reflect central adaptations in oxygen transport and peripheral improvements in muscular oxygen utilization (32, 34). Caffeine supplementation (3 mg·kg−1) before SIT produced greater VO2peak enhancements, suggesting added benefits for this population. Possible mechanisms include caffeine metabolism to theobromine, a vasodilator that increases oxygen and nutrient delivery to brain and muscle (14, 35), and paraxanthine, which elevates circulating glycerin and fatty acids as energy substrates (36). Caffeine may also promote calcium release, activating glycogen phosphorylase B and enabling aerobic exercise at lower perceived exertion and fatigue (37) (Figure 4). These mechanisms remain plausible literature-based explanations, as they were not directly measured in this study and should be examined in future to clarify the exact effects of biochemical responses to caffeine supplementation and short SIT intervention in overweight and obese women.

Both Caffeine and Placebo groups experienced beneficial transfer effects from short SIT, improving lipid profiles in overweight and obese women after 12 weeks. These findings align with previous reports on the positive effects of various HIIT formats on lipid profile management (4, 34). Such improvements may stem from increased mRNA expression of PPARγ and PGC-1α in muscle and adipose tissue (38), which, together with SIT-induced aerobic metabolic conditioning, enhances lipoprotein lipase activity to stimulate oxidative pathways and modify lipid profiles (39). Caffeine ingestion before SIT produced no further lipid-related adaptations, consistent with earlier research (40). This absence of additional caffeine-induced lipid changes suggests that caffeine’s ergogenic effects may not extend to lipid metabolism under normal baseline lipid conditions (41). One possible explanation for the lack of further changes could be the participants’ normal lipid levels, insufficient supplementation duration, or the specific demands of short SIT. This underscores the need for further research in populations with dyslipidemia to assess whether caffeine influences lipid profiles in a clinically meaningful way.

Both Caffeine and Placebo groups showed significant reductions in fasting glucose after 12 weeks, consistent with evidence that HIIT improves glucose metabolism in overweight and obese individuals (4, 42). Such benefits likely involve increased glucose transport to active muscle fibers, activation of protein kinase B, and greater GLUT4 expression, which together promote muscle glucose uptake for energy production (43). Adaptive responses in adipokines—higher irisin and adiponectin and lower leptin—also paralleled improved glucose control (44). The Caffeine group (3 mg·kg−1 before SIT) achieved trivial to small greater improvements in fasting glucose and adipokine profiles than Placebo (45, 46). Although the observed differences reached statistical significance, their practical or clinical importance for individuals who do not have underlying metabolic disorders is likely minimal. Such findings, therefore, should be interpreted cautiously and warrant further investigation in future studies—particularly in populations at risk or with existing metabolic impairments—to determine whether these adaptations translate into meaningful health benefits.

**Figure 5:**
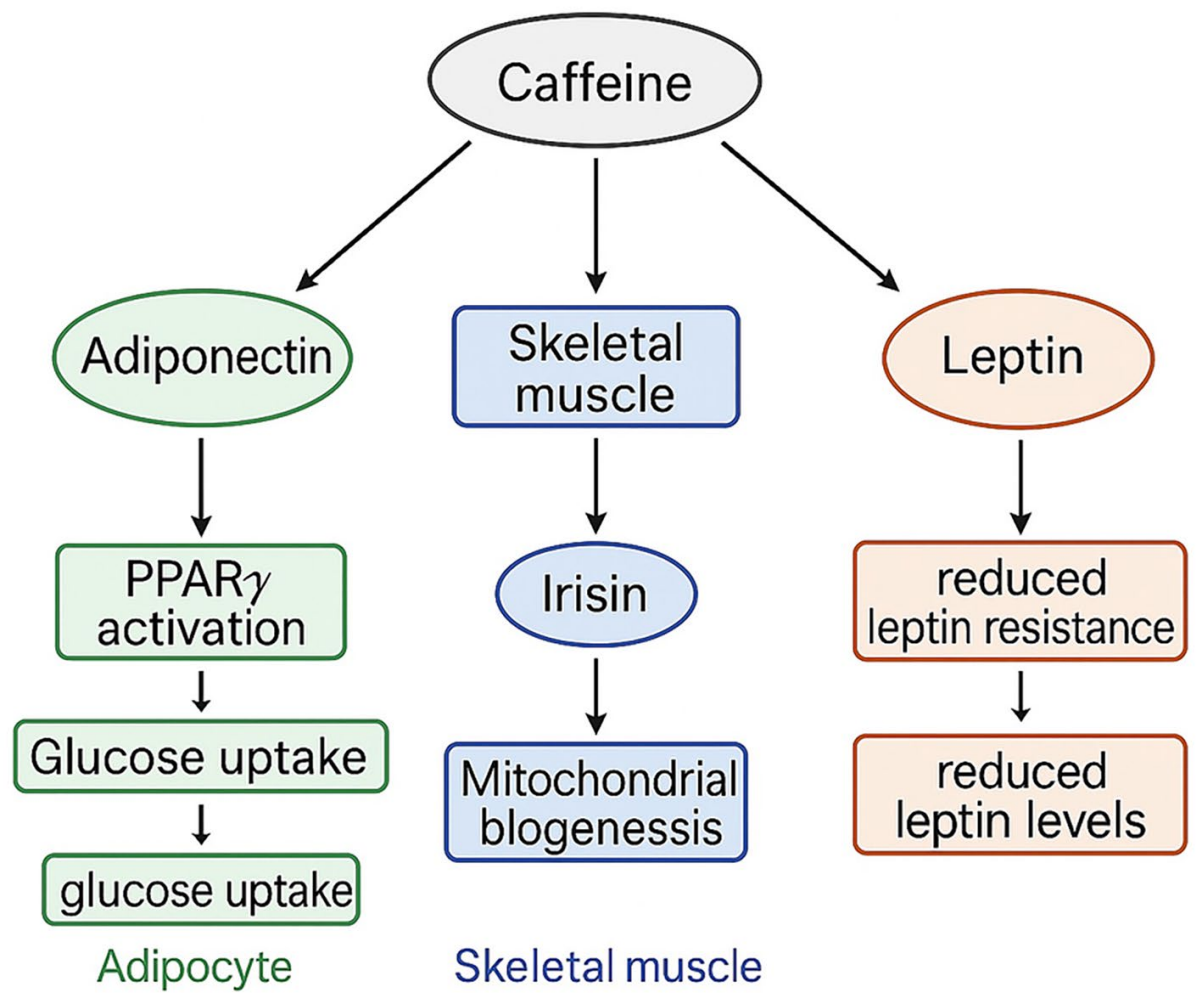
The mechanisms of caffeine in glucose regulation.

The mechanisms underlying caffeine’s influence on adipokine adaptations may be linked to its direct effects on adipocytes (46–48). Evidence suggests that caffeine can upregulate peroxisome proliferator-activated receptor γ (PPARγ), a key regulator of adipocyte differentiation and maintenance, thereby facilitating increased adiponectin production (46, 47). Caffeine also activates calcium signaling pathways and PGC-1α in skeletal muscle, stimulating irisin release (47). Furthermore, caffeine intake may reduce leptin resistance and enhance leptin sensitivity, contributing to the observed decrease in leptin levels over the 12-week intervention (49, 50). Collectively, these pathways suggest that participants consuming 3 mg·kg−1 caffeine prior to each SIT session experienced cellular adaptations leading to higher adiponectin and irisin levels, coupled with lower leptin, ultimately improving glucose regulation via GLUT4 activation (45, 49) (Figure 5). These interpretations are based on prior literature and should be regarded as plausible mechanistic hypotheses requiring confirmation through direct biochemical measurements in future studies.

This research employed an innovative approach for analyzing data to gain insights into individual responses to caffeine supplementation and short SIT. Rather than simply presenting mean values with SDs and ESs, employing IRs in ∆% and CVs allows for a clearer understanding of how participants responded to the training and supplementation, as well as the uniformity of these responses among subjects. To our knowledge, previous studies on SIT or caffeine supplementation in similar populations have not computed or reported IRs and CVs for adaptive outcomes, making this a novel methodological contribution. The findings of this study indicate that caffeine supplementation not only led to greater reductions in fat mass, fasting glucose levels, and increases in irisin and adiponectin, but also resulted in lower levels of leptin, and the caffeine group exhibited more consistent adaptations, as evidenced by lower IRs and CVs compared to the placebo group. Lower CVs and IRs indicate that participants responded more uniformly to caffeine supplementation combined with SIT, suggesting greater predictability of performance and physiological adaptations and reduced variability in individual responses — a characteristic of practical relevance for practitioners working with obese and overweight women to optimize training outcomes in these populations. This aligns with the broader literature on individual variability in responsiveness to exercise and nutritional supplementation, while extending it by quantifying response consistency with IRs and CVs. These insights may have practical applications for tailoring interventions to improve individual responsiveness. Consequently, the combination of short SIT with caffeine supplementation elicited more consistent adaptations than those observed with placebo in overweight and obese women.

This study has several limitations that should be considered when interpreting the findings. First, the study’s sample size was modest (*n* = 10), which may limit the generalizability of the findings. Nonetheless, an a priori power analysis confirmed that this number was sufficient to achieve the desired statistical power (17). Additionally, the cohort was restricted to young, sedentary, overweight or obese women, which limits the applicability of the findings to other populations such as men, older adults, trained individuals, or those with different body composition or health status. Although the placebo was designed to be indistinguishable from the active intervention in terms of appearance, texture, and taste, we did not conduct a formal assessment of blinding efficacy (e.g., asking participants to guess their group allocation). The absence of such evaluation limits our ability to confirm the success of blinding and may introduce the possibility of bias in the interpretation of outcomes. Furthermore, the absence of direct biochemical and neuromuscular assessments related to the proposed mechanisms underlying the observed changes prevents definitive conclusions about the physiological pathways involved. Future research with larger, more diverse populations and comprehensive mechanistic testing is warranted to confirm and extend these findings.

## 5 Conclusion

Our findings suggest that short SIT is an effective approach for eliciting physiological adaptations in women with overweight and obesity. When combined with caffeine supplementation, these adaptations may be further enhanced, including possible reductions in fat mass, gains in strength, improvements in cardiorespiratory fitness, and more favorable regulation of fasting glucose and adipokine profiles. In light of these outcomes, caffeine supplementation alongside training appears to promote more consistent and potentially greater improvements in performance, body composition, and other physiological markers than placebo, indicating its promise as a targeted ergogenic strategy in similar populations and training modalities, while acknowledging that the modest effect sizes and limited sample warrant cautious interpretation.

## Data Availability

The raw data supporting the conclusions of this article will be made available by the authors, without undue reservation.
